# A genome assembly and transcriptome atlas of the inbred Babraham pig to illuminate porcine immunogenetic variation

**DOI:** 10.1007/s00251-024-01355-7

**Published:** 2024-09-19

**Authors:** John C. Schwartz, Colin P. Farrell, Graham Freimanis, Andrew K. Sewell, John D. Phillips, John A. Hammond

**Affiliations:** 1 The Pirbright Institute, Ash Road, Woking, GU24 0NF UK; 2 Division of Hematology, University of Utah School of Medicine, Salt Lake City, UT 84112 USA; 3 Department of Molecular, Cell and Developmental Biology, University of California Los Angeles, Los Angeles, CA 90095 USA; 4 Division of Infection and Immunity, Cardiff University School of Medicine, Cardiff, CF14 4XN UK

**Keywords:** Major histocompatibility complex, B cell receptor, T cell receptor, Leukocyte receptor, Natural killer cell receptor

## Abstract

The inbred Babraham pig serves as a valuable biomedical model for research due to its high level of homozygosity, including in the major histocompatibility complex (MHC) loci and likely other important immune-related gene complexes, which are generally highly diverse in outbred populations. As the ability to control for this diversity using inbred organisms is of great utility, we sought to improve this resource by generating a long-read whole genome assembly and transcriptome atlas of a Babraham pig. The genome was de novo assembled using PacBio long reads and error-corrected using Illumina short reads. Assembled contigs were then mapped to the porcine reference assembly, Sscrofa11.1, to generate chromosome-level scaffolds. The resulting TPI_Babraham_pig_v1 assembly is nearly as contiguous as Sscrofa11.1 with a contig N50 of 34.95 Mb and contig L50 of 23. The remaining sequence gaps are generally the result of poor assembly across large and highly repetitive regions such as the centromeres and tandemly duplicated gene families, including immune-related gene complexes, that often vary in gene content between haplotypes. We also further confirm homozygosity across the Babraham MHC and characterize the allele content and tissue expression of several other immune-related gene complexes, including the antibody and T cell receptor loci, the natural killer complex, and the leukocyte receptor complex. The Babraham pig genome assembly provides an alternate highly contiguous porcine genome assembly as a resource for the livestock genomics community. The assembly will also aid biomedical and veterinary research that utilizes this animal model such as when controlling for genetic variation is critical.

## Introduction

Pigs (*Sus scrofa*) are vital to both biomedical research and the production of pork, the most extensively consumed meat product worldwide (USDA 2022). The anatomical and physiological similarities with humans make pigs an excellent model of human disease, such as for tuberculosis or influenza (Bolin et al. 1997; Groenen et al. 2012; Holzer et al. 2021; Lunney et al. 2021; Perleberg et al. 2018), and their similar organ sizes make pigs ideally suited as a source of organs for xenotransplantation (Ekser et al. 2017; Lunney 2007; Lunney et al. 2021). Furthermore, pigs continue to face ongoing threats from African swine fever and other diseases, especially in east Asia, and research into effectively controlling these diseases is important for global food security and for improving animal welfare (Kedkovid et al. 2020).

The pig reference genome assembly (Groenen et al. 2012; Warr et al. 2020) has greatly contributed to our understanding of porcine immunology (Dawson et al. 2013; Hammer et al. 2020; Le Page et al. 2021; Linguiti et al. 2022; Massari et al. 2018; Morgan et al. 2018; Schwartz et al. 2017; Schwartz and Hammond 2018; Zhang et al. 2020) and enhanced the pig’s role as a model of disease (Burkard et al. 2018; Groenen et al. 2012; Nicholls et al. 2016; Perleberg et al. 2018; Whitworth et al. 2014) and as a potential xenotransplantation donor (Niu et al. 2021, 2017). Improvements in long-read sequencing technologies and whole genome assembly techniques within the last decade have resulted in greatly improved mammalian genome assemblies, with contig lengths now approaching that of whole chromosomes (Bickhart et al. 2017; Bredemeyer et al. 2021; Koren et al. 2018; Low et al. 2020; Rice et al. 2020; Rosen et al. 2020; Warr et al. 2020). Among these endeavors, the pig reference genome was recently updated with Illumina paired-end reads and Pacific Biosciences (PacBio) single-molecule real-time sequencing reads for gap filling. While these sequences were generated using genomic DNA from the same purebred Duroc sow used for the earlier pig reference assembly, additional Y-chromosome sequence from a male individual was incorporated into the current assembly, Sscrofa11.1 (Warr et al. 2020). Current and future efforts to generate gapless telomere-to-telomere (T2T) assemblies promise to revolutionize the field of genomics (Kalbfleisch et al. 2024; Nurk et al. 2022).

As an animal model with a defined genetic background and limited heterozygosity, the inbred Babraham pig holds great potential for the research community, and several recent studies have used it to investigate immune responses in the pig while leveraging the breed’s minimal genomic variability (Baratelli et al. 2020; Edmans et al. 2021; Lefevre et al. 2012; Martini et al. 2021; Muir et al. 2024; Nicholls et al. 2016, 2012; Tungatt et al. 2018). The breed was initially developed from commercial Large White pigs at The Babraham Institute (Cambridge, UK) in the 1970s as a model organism and is currently the only extant large inbred pig breed available for research (Schwartz et al. 2018). Individuals were selectively bred to display the least amount of cross-rejection after multiple skin grafts, eventually producing animals with full cross-tolerance (Signer et al. 1999). Such graft tolerance suggested homozygosity across the major histocompatibility complex (MHC), which was later confirmed (Nicholls et al. 2016; Schwartz et al. 2018; Signer et al. 1999), and restriction fragment length polymorphism patterning also further indicated a level of inbreeding comparable to that of inbred mice (Signer et al. 1999).

Pigs are natural hosts of influenza A virus (IAV) and infection represents a substantial problem for the agricultural industry (Brown 2000). Pigs can be infected with human and bird forms of IAV which can recombine with swine virus to generate antigenic shift and create dangerous pandemic strains (Ito et al. 1998; Ma et al. 2009). The Babraham pig has become an important model for understanding human influenza infection and for the development of new vaccines against IAV and other swine viruses (Lefevre et al. 2012; Rajao and Vincent 2015). The dominant influenza peptide antigens presented by Babraham MHC molecules (also known as swine leukocyte antigen (SLA)) have been described, and peptide-SLA multimers have been used to study spatial, temporal, and molecular dynamics of swine flu-specific CD8 + tissue-resident T cells (Martini et al. 2022) and assess responses to IAV vaccines (Goatley et al. 2022; Martini et al. 2021; Muir et al. 2024). The absence of detailed architectural knowledge of the Babraham antigen receptor loci remains the major bottleneck in the Babraham model of viral infection. We set out to bridge this critical knowledge gap to bring this swine model to the level of understanding available in human or laboratory mice.

To improve the Babraham pig as a resource for transcriptomic and immunological studies, we utilized PacBio long-read sequencing and assembly, Illumina short-read error correction, and reference-guided scaffolding to generate a highly contiguous genome assembly of the inbred Babraham pig that is almost as contiguous as the reference assembly. As immune-related gene complexes often contain many tandemly duplicated paralogous genes that can be highly similar in sequence and of variable gene content, their repetitiveness often disrupts genome assemblies (Bickhart et al. 2017; Rosen et al. 2020). We therefore specifically investigated the homozygosity, contiguity, gene content, and tissue expression of several highly variable regions that are important in lymphocyte immunobiology, including the B cell (IGH, IGK, and IGL) and T cell receptor (TRB, TRG, TRA/TRD) loci, MHC class I and class II, the natural killer complex (NKC), and the leukocyte receptor complex (LRC).

## Materials and methods

### Animal use and ethics statement

A representative adult male Babraham pig (animal ID: P18-11073), whose parents were half-siblings, was culled from the herd managed by The Pirbright Institute and held at the Animal and Plant Health Agency (APHA; Addlestone, UK) in the context of routine herd maintenance. This was approved by both The Pirbright Institute Animal Welfare and Ethical Review Body and the APHA Animal Welfare and Ethics Committee under the authority of the UK Home Office establishment license for APHA (X16E7B018) in accordance with the UK Animals (Scientific Procedures) Act 1986.

### Genomic DNA purification and sequencing

Tissue from the frontal lobe of the cerebral cortex was selected for whole genome sequencing because it lacks immune cells with rearranging receptors (i.e., B cells and T cells), which may complicate assembly efforts across these respective genetic loci. A sample of the tissue was provided to the University of Utah Core Research Facilities (Salt Lake City, Utah) on dry ice for high molecular weight genomic DNA purification and sequencing. For genome assembly, long-read sequencing was performed using the PacBio Sequel II platform in continuous long read (CLR) mode with the SMRTbell Express Template Prep Kit 2.0 (Pacific Biosciences of California, Inc., Menlo Park, California). This resulted in 11,141,834 reads with an average read length of 12,552 bp (~ 57 × coverage). For error correction, short-read sequences were generated using the Illumina TruSeq DNA PCR-Free library preparation kit and the Illumina NovaSeq 6000 platform (Illumina, Inc., San Diego, CA) which resulted in 415,666,795 paired-end 150 bp reads (~ 51 × coverage).

Additional genomic DNA was prepared from fibroblast cells collected from a different male Babraham pig which were archived at The Pirbright Institute circa 2015. Approximately 3 × 10^7^ cells were resuspended in 5 ml of PBS and lysed with 25 ml lysis buffer (140 mM NH_4_Cl and 17 mM Tris–HCl, pH 7.4). The resulting pellet was then resuspended in 9 ml (10 mM Tris–HCl, 400 mM NaCl, 2 mM EDTA, pH 8.0) and digested for 1 h at 37 °C after the addition of 10% sodium dodecyl sulfate (600 µl) and 100 mg/ml RNase A (13 µl). Nucleases were then inactivated with the addition of 20 mg/ml Proteinase K (100 µl) for 8 h. High molecular weight genomic DNA was then precipitated by adding 6 M NaCl (3 ml), centrifuging, treating the supernatant with two volumes (~ 26 ml) of 100% ethanol, and centrifuging again to produce a DNA pellet that was further purified using 80% ethanol. The final pellet was resuspended in 0.1 × TE buffer (1 mM Tris and 0.1 mM EDTA, pH 8.0) and quantified using a TapeStation 4150 (Agilent Technologies, Inc., Santa Clara, CA). As above, DNA was provided to the University of Utah Core Research Facilities for Illumina TruSeq DNA PCR-Free library preparation and sequencing using a HiSeq 2500 which generated 278,898,802 paired-end 125 bp reads (approximately 28 × coverage).

### Genome assembly and error correction

The PacBio CLR sequencing reads were *de novo* assembled into contigs and scaffolded using Flye, v2.5 (Kolmogorov et al. 2019) with parameters set to –asm-coverage 30 -t 30 and error-corrected using Pilon (version 1.24) (Walker et al. 2014) and the P18-11073 Illumina sequences. The error-corrected contigs/scaffolds were then mapped to the Sscrofa11.1 chromosomal assembly (GenBank: GCA_000003025.6) using Minimap2 (Li 2018). This mapping was used to order and orient the Babraham contigs into chromosomes, in which the *de novo* assembled contigs and scaffolds were separated by a span of 100 N’s. Orientation and identity were confirmed by mapping these chromosomal assemblies back to Sscrofa11.1 using Minimap2 with the preset parameter -x asm5 for long assembly to reference mapping with up to 5% sequence divergence (Li 2018). The Minimap2 output in pairwise mapping format (PAF) was then visualized for each chromosome in R (v3.4.1) using dotPlotly with parameters set to -m 100 -q 50000 (Poorten n.d.). The 1034 unplaced contigs were screened for contaminating sequence using Kraken (version 1.1.1) and the complete Kraken database including viral, bacterial, and fungal sequence (Wood and Salzberg 2014). This flagged 378 contigs as potentially containing contaminating viral or bacterial sequence. However, all except two of these successfully mapped to Sscrofa11.1 using Minimap2, indicating that the Kraken hits were false positives. The remaining two unmapped contigs fully contained relatively simple repeats (i.e., A(C_n_) _n_ and (TTTAAC) _n_). Thus, all 1034 unplaced contigs were retained in the final assembly.

### Analysis of heterozygosity

Short-read whole genome sequencing reads were mapped to Sscrofa11.1 using the Burrows-Wheeler Aligner (BWA; version 0.7.12) (Li and Durbin 2009). For the Babrahams, this included both the 4.16 × 10^8^ reads from P18-11073 and the 2.79 × 10^8^ reads from the primary Babraham fibroblast cells described above. For the Duroc (i.e., “TJ Tabasco”), FASTQ files collectively containing approximately 3.74 × 10^8^ Illumina HiSeq 150 bp paired-end sequencing reads (~ 46 × coverage) were acquired from BioProject accession PRJEB9115. Sequences for MARC1423004, the individual used to generate the USMARCv1.0 assembly, were acquired from the 16 NextSeq 500 runs archived within BioProject accession PRJNA392765 and totaled 1.79 × 10^9^ paired-end 150 bp sequencing reads (~ 220 × coverage). Variant sites were identified using SAMtools (version 1.2) and BCFtools (version 1.3.1) (Li 2011a; Li et al. 2009), and the resulting VCF files were indexed with Tabix (version 1.10.2–45-gb22e03d) (Li 2011b). Only heterozygous sites with a Phred-scaled QUAL score ≥ 30 were considered for further analyses. For the Babraham and MARC1423004 sequences, the total number of heterozygous sites (ALT/REF and ALT1/ALT2) was summed within each 200 kb window. For the Duroc, any ALT1/ALT2 sites would be the result of mapping error, so only the total number of ALT/REF sites was summed for each 200 kb window. Heterozygosity across the genome was then visualized using Gitools version 2.3.1 (Perez-Llamas and Lopez-Bigas 2011).

### Telomeric and centromeric repeats

Telomeric repeats were identified by searching for repeat sequences containing exact matches of at least three tandem hexamers of either TTAGGG or CCCTAA using Tandem Repeats Finder (TRF) version 4.09 (Benson 1999). The number of hexamers within each identified repeat was summed and visualized across each chromosome using Gitools version 2.3.1 (Perez-Llamas and Lopez-Bigas 2011) and a window size of 200 kb. Output from TRF was also used to identify large (i.e., 10 kb (chr15) to 552 kb (chr2)) centromeric repeat regions in the expected chromosomal locations based on previous analyses (Hansen 1977; Warr et al. 2020). Gitools was also used to visualize these centromeric repeat regions within each chromosomal assembly.

### Annotation of immune-related gene complexes

Assembled chromosomes and unplaced contigs were queried using both the basic local alignment search tool (BLAST) (Altschul et al. 1990) for genes of interest within the natural killer complex (NKC), leukocyte receptor complex (LRC), major histocompatibility complex (MHC), and T cell and B cell receptor loci using previously reported characterizations or from IPD-MHC (Eguchi-Ogawa et al. 2012; Hammer et al. 2020; Le Page et al. 2021; Lunney et al. 2009; Maccari et al. 2017, 2020; Massari et al. 2018; Schwartz et al. 2017, 2018, 2012a, b; Schwartz and Hammond 2018), which the Babraham was compared to. This was aided with the use of the conserved domain search tool (Marchler-Bauer and Bryant 2004; Marchler-Bauer et al. 2015) to help identify additional genes and gene fragments. Exons were manually annotated within the chromosomal assemblies using Artemis (version 17.0.1) (Rutherford et al. 2000). Pig Iso-Seq data (BioProject: PRJNA351265) derived from multiple tissues (i.e., small intestine, pituitary, spleen, diaphragm, longissimus muscle, brain, hypothalamus, thymus, and liver) were also used to determine splice variation and confirm exon boundaries within the Babraham genome assembly (Beiki et al. 2019). MHC alleles were named based on their identity to known alleles within IPD-MHC (Maccari et al. 2017, 2020). Recurrence plot comparisons of gene loci between the Babraham and Sscrofa11.1 assemblies were generated using Dotter (version 4.44.1) (Sonnhammer and Durbin 1995).

### Transcriptome sequencing and analyses

Twelve tissue samples (frontal cortex (brain), liver, kidney, spleen, lung, tonsil, bronchial lymph node, mesenteric lymph node, Peyer’s patch, thymus, heart, and testes) were collected from pig P18-11073 and stored in RNAlater (Thermo Fisher Scientific Inc., Waltham, MA) for subsequent transcriptomic analyses. Processed in triplicate, these were disrupted and homogenized in RLT lysis buffer containing β-mercaptoethanol (Qiagen, GmbH, Hilden, Germany) using a rotor–stator homogenizer. Peripheral blood was collected into tubes containing 10 IU heparin sodium per ml of blood (Wockhardt UK Ltd., Wrexham, UK). Mononuclear cells (PBMCs) were subsequently isolated using Histopaque-1083 following the manufacturer’s instructions (Sigma-Aldrich, St. Louis, MO) and aliquoted into samples containing approximately 2 × 10^7^ cells each. Triplicate PBMC samples were disrupted in RLT lysis buffer containing β-mercaptoethanol and homogenized using QIAshredder spin columns (Qiagen). RNA was extracted and purified from all PBMC and tissue homogenates using the Qiagen RNeasy Plus Mini Kit which includes a genomic DNA removal step. RNA quality (i.e. 260:280 ratio) was assessed using a NanoDrop 1000 spectrophotometer (Thermo Fisher Scientific) and quantified using both a Qubit 2.0 fluorometer with the RNA High Sensitivity Assay Kit (Invitrogen, a subsidiary of Thermo Fisher Scientific Inc.) and a Bioanalyzer 2100 with the RNA 6000 Nano Kit (Agilent Technologies). Sequencing libraries were prepared using the NEBNext Ultra II Directional RNA Library Prep Kit for Illumina using random priming (New England Biolabs, Ipswich, MA). In total, approximately 1.73 × 10^9^ single-end 150 bp sequencing reads were generated from all 39 samples (i.e., 13 tissues × 3 replicates) using the Illumina NextSeq 550 platform at The Pirbright Institute. All transcriptomic sequencing reads were deposited in the National Center for Biotechnology Information (NCBI) Sequence Read Archive under BioProject accession PRJNA1098952.

Transcriptomic reads from 39 FASTQ files representing the 13 tissues in triplicate were each mapped to both the Sscrofa11.1 (GenBank: GCA_000003025.6) and TPI_Babraham_pig_v1 (GenBank: GCA_031225015.1) genome assemblies using STAR (version 2.7.1a) (Dobin et al. 2013). The output BAM files were sorted and indexed using samtools sort and samtools index (version 1.2), respectively, and overall coverage depth was determined using samtools depth (Li et al. 2009). Because a genome-wide annotation does not yet exist for the TPI_Babraham_pig_v1 assembly, we used Sscrofa11.1 as a proxy to generate and normalize read counts for the whole transcriptome. Reads that were aligned to Sscrofa11.1 were filtered for alignments to the publicly available annotated transcriptome using bedtools intersect (version 2.27.1) (Quinlan and Hall 2010) and the Sscrofa11.1 GTF file obtained from Ensembl release 103 (Howe et al. 2021). Raw total read counts and reads per kilobase (RPK) were then calculated for each transcript using either the output for the whole transcriptome from featureCounts (version 1.6.3) (Liao et al. 2014) or the output for the manually annotated genes of interest from bedtools coverage. Normalized gene-length corrected trimmed mean of M-values (GeTMM) (Smid et al. 2018) were calculated from the RPK values using the edgeR package (version 3.20.9) (Robinson et al. 2010) within R (version 4.2.2) and visualized using Gitools version 2.3.1 (Perez-Llamas and Lopez-Bigas 2011).

## Results

### A highly contiguous de novo assembly of the Babraham pig genome

Approximately 1.11 × 10^7^ PacBio Sequel II CLR sequencing reads with an average read length of 12,552 bp and read N50 of 22,299 bp were generated, amounting to approximately a 57-fold coverage of the porcine genome. Reads were *de novo* assembled into contigs and scaffolds using Flye (v2.5) (Kolmogorov et al. 2019) and error-corrected using Pilon (version 1.24) (Walker et al. 2014) and approximately 51-fold coverage of Illumina (2 × 150 bp) reads from the same animal. Contigs were then screened for contaminating sequence using Kraken (version 1.1.1) (Wood and Salzberg 2014). However, this did not identify any contamination, and all contigs either successfully mapped to Sscrofa11.1 or contained simple repeats. The resulting assembly consists of 2447 Mb across 1391 contigs with a contig N50 of 34.95 Mb and contig L50 of 23. The assembled contigs and scaffolds were mapped to the pig reference genome assembly, Sscrofa11.1 (Warr et al. 2020), to generate a chromosome-level assembly (Table 1). This resulted in a placement of 357 contigs spanning 2408 Mb across the 18 autosomes, Chr X, Chr Y, and the mitochondrial chromosome. The remaining 1034 unplaced contigs, comprising 40 Mb, were generally much smaller with a contig N50 of 150 kb and are likely unplaced Chr Y sequence and alternative haplotype sequences.

**Table 1:** Chromosome-level assembly statistics for Sscrofa11.1, USMARCv1.0, and TPI_Babraham_pig_v1

Chr	Sscrofa11.1			USMARCv1.0			TPI_Babraham_pig_v1		
	Ungapped length (bp)	Number of contigs	Contig N50 (bp)	Ungapped length (bp)	Number of contigs	Contig N50 (bp)	Ungapped length (bp)	Number of contigs	Contig N50 (bp)
1	274,330,132	5	90,927,422	268,199,312	66	6,467,034	278,785,404	15	60,958,165
2	151,800,670	7	87,417,173	141,039,314	37	8,371,877	150,500,149	53	36,467,456
3	132,648,513	9	73,254,198	128,651,370	35	6,713,922	133,369,682	10	21,452,984
4	130,870,669	4	100,518,328	128,001,252	30	11,944,967	131,187,407	12	34,019,294
5	104,375,107	13	21,111,347	98,929,882	27	6,762,634	107,091,043	11	17,630,615
6	170,419,461	11	18,397,423	160,955,110	41	7,899,165	170,189,100	18	20,338,316
7	121,743,199	12	29,790,190	119,961,677	18	29,051,871	125,629,992	26	22,695,760
8	138,865,937	6	72,677,949	135,855,389	33	6,513,734	139,153,575	15	76,299,722
9	139,511,883	3	133,627,600	135,417,841	27	10,245,400	139,228,276	15	41,712,811
10	69,257,333	4	44,332,889	68,415,272	22	5,169,423	70,201,652	9	10,942,133
11	79,119,678	5	19,474,953	77,145,484	16	6,656,154	79,744,139	5	29,255,778
12	61,500,128	4	45,299,297	56,950,340	19	4,652,640	61,381,331	12	18,011,077
13	208,234,567	11	24,026,255	199,810,805	50	7,414,089	208,105,327	14	48,808,734
14	141,755,246	3	130,192,676	139,163,928	16	38,681,723	141,998,002	15	34,948,847
15	140,362,525	4	38,129,723	136,633,314	30	7,208,255	140,491,208	15	81,360,748
16	79,944,280	1	79,944,280	77,627,177	24	5,327,307	80,090,520	6	44,193,870
17	63,343,681	8	48,231,277	62,541,674	14	8,416,923	63,352,208	14	48,195,611
18	55,982,971	1	55,982,971	55,717,653	7	30,967,442	55,898,280	2	48,439,141
X	125,778,901	11	16,842,758	99,540,920	159	960,692	125,650,420	63	4,139,144
Y	17,132,043	413	66,937	2,610,849	9	255,686	5,552,120	26	635,153
M	16,613	1	16,613	16,760	1	16,760	16,701	1	16,701
Unplaced	65,054,210	583	250,081	329,944,915	14,137	23,975	39,999,133	1034	149,592
Total	2,472,047,747	1119	48,231,277	2,623,130,238	14,818	6,372,407	2,447,615,669	1391	34,948,847

**Fig. 1:**
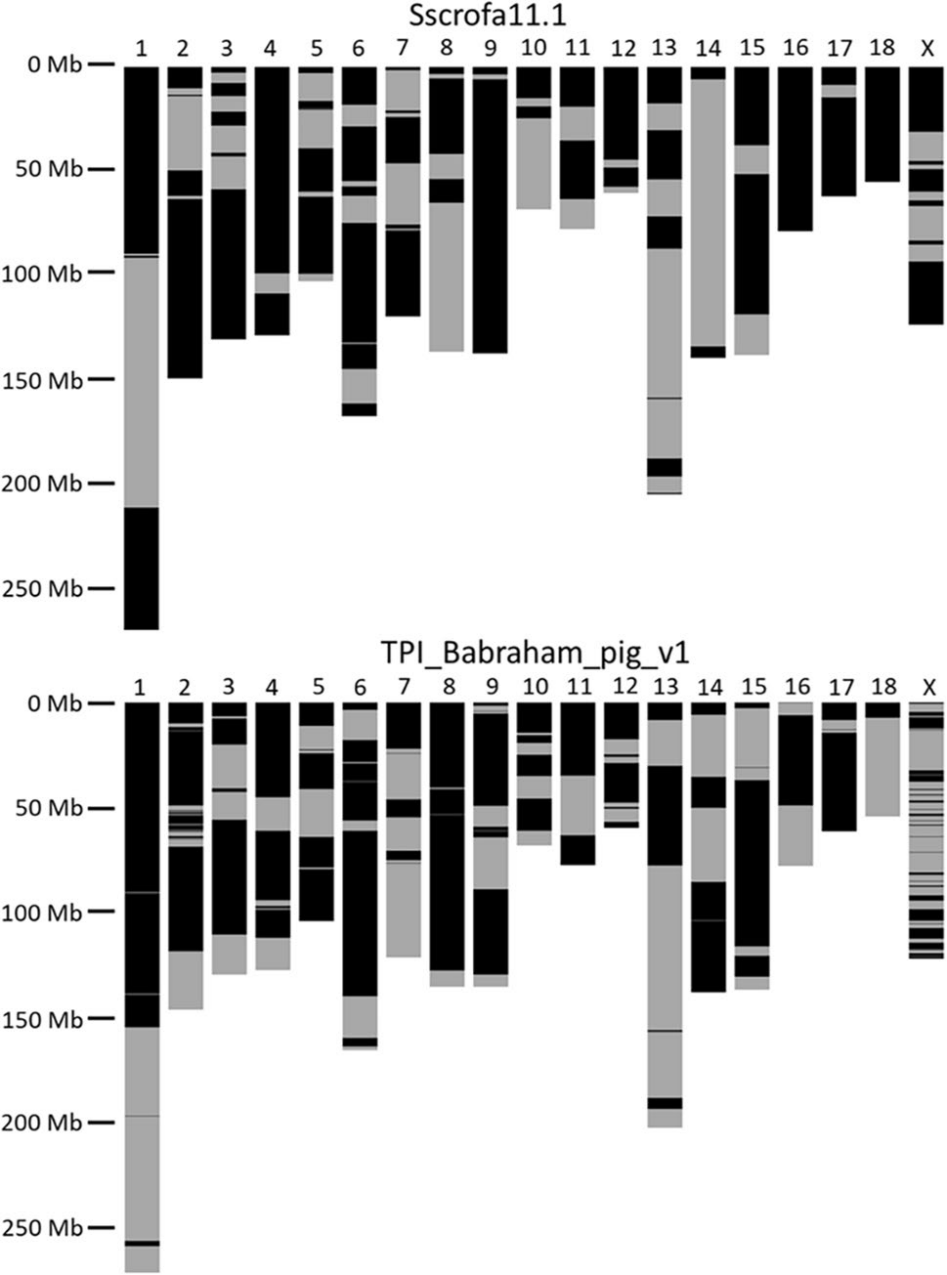
Contiguity of autosomal and Chr X assemblies. Contigs are indicated by alternating dark and light bands. Contigs smaller than 100 kb are not shown as they are too small to reasonably resolve

**Fig. 2:**
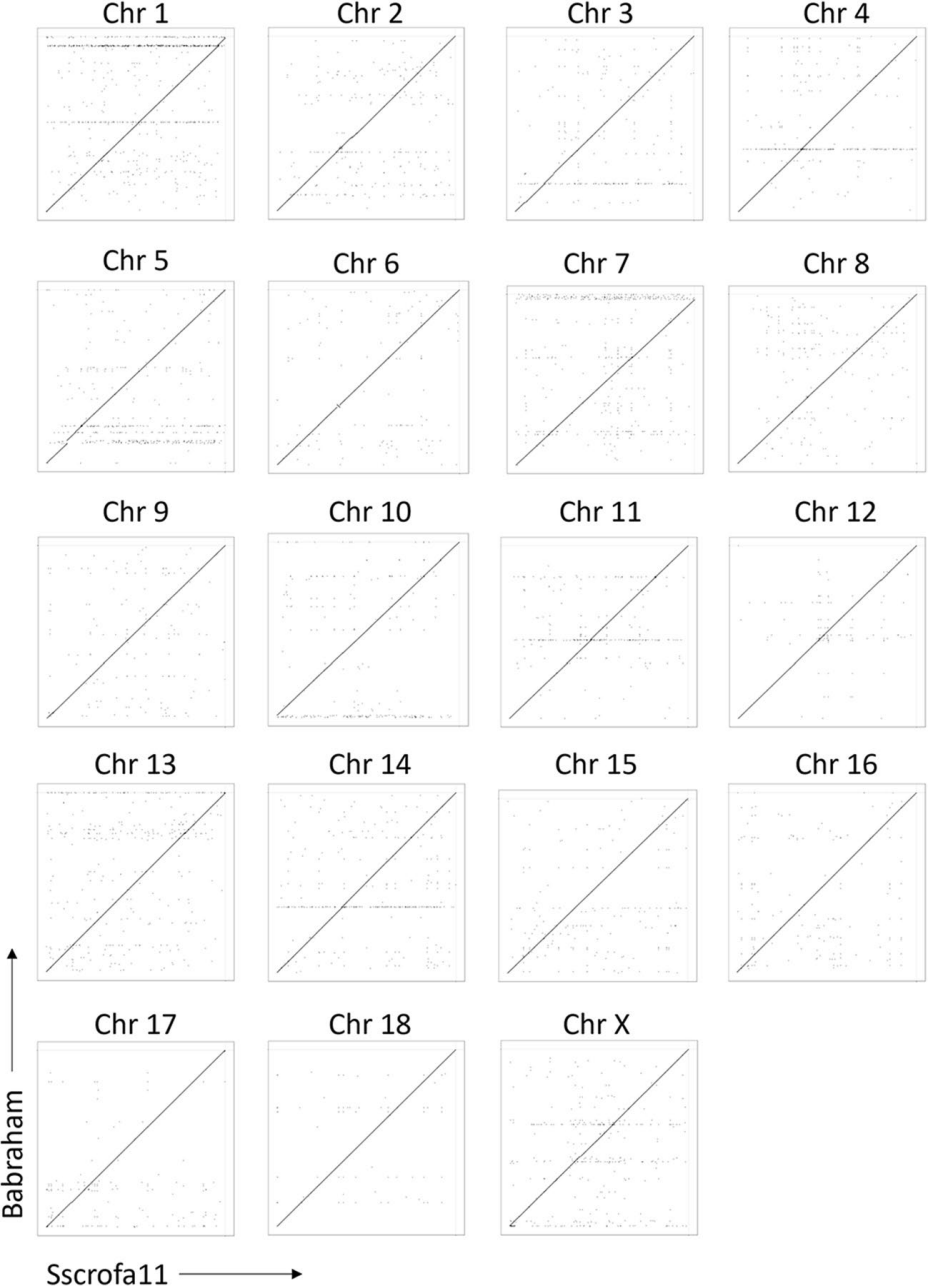
Consistency of orientation and contig ordering between assemblies. Recurrence plot comparisons of TPI_Babraham_pig_v1 (*vertical axes*) and Sscrofa11.1 (*horizontal axes*) autosomal and Chr X assemblies

The contiguity across the autosomes and Chr X is comparable between the Babraham and the Sscrofa11.1 assemblies (Fig. 1). The allosomes, Chr X and Chr Y, are the least contiguous, and while the former is approximately the same length in the two assemblies, the Babraham Chr Y assembly is only 32% the total length of Chr Y in Sscrofa11.1, indicating that a large proportion of Chr Y likely remains unplaced in the Babraham assembly. Fifty of the unplaced contigs mapped at least partially to the Chr Y assembly of Sscrofa11.1; however, the combined size of these contigs totaled only 2.4 Mb, indicating that a considerable amount of Chr Y remains unaccounted for. Sequence orientation and contig order were further confirmed for the autosomes and Chr X by mapping their assemblies back to Sscrofa11.1 (Fig. 2).

### Centromeric and telomeric repeats disrupt the sequence contiguity of the assembly

**Fig. 3:**
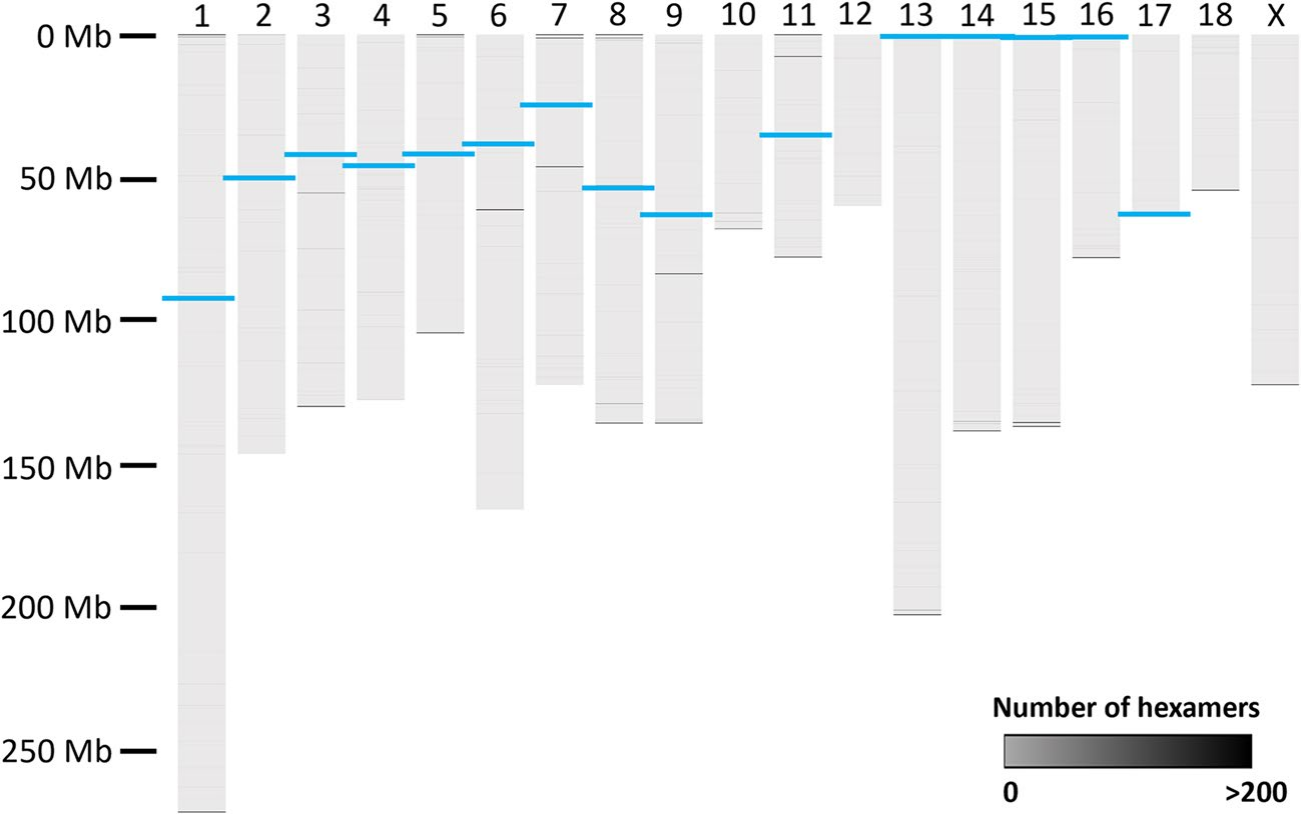
Centromeric and telomeric repeats in the TPI_Babraham_pig_v1 assembly. Repeats of telomeric hexamers are shown as *grey* bars of variable intensity. The positions of the centromeres are shown as thicker *blue* bars

We next attempted to determine the degree of contiguity loss due to large and repetitive sequences, specifically the telomeres and centromeres, as these are likely to disrupt assembly contiguity. We detected centromeric repeats in the expected locations for all but three autosomes (Chr 10, Chr 12, and Chr 18) and Chr X, in which the centromeres were not identified (Fig. 3). Of the remaining, all are disrupted by either a sequence gap (Chr 1 to Chr 12) or truncated, as is the case for the telocentric chromosomes (Chr 13 to Chr 18). Thus, not unexpectedly, the large repeat structures associated with the centromeres were problematic for the contiguous assembly of the genome. Furthermore, as noted for the Sscrofa11.1 assembly (Warr et al. 2020), the centromere of Chr 17 is found on the opposite end of the assembly as conventionally presented. However, to conform to the published reference assembly, we retained this reversed orientation for Chr 17.

Telomeric repeats were identified at both terminal ends of four chromosomes (Chr 1, Chr 5, Chr 8, and Chr 11), at one end of ten chromosomes (Chr 3, Chr 7, Chr 9, Chr 10, Chr 13 to Chr 16, Chr 18, and Chr X, including five of the six telocentric chromosomes), and at neither end of five chromosomes (Chr 2, Chr 4, Chr 6, Chr 12, and Chr 17), indicating likely truncated assemblies at the ends of some of the chromosomes. Internal telomeric repeats containing > 90 hexamers were also identified on Chr 3, Chr 6, Chr 7, Chr 9, and Chr 11 (Fig. 3) and are likely the remnants of ancestral chromosomal fusion events (Kumar et al. 2017; Thomsen et al. 1996). All except one of these internal repeats is contiguously assembled; having 6521 assembled hexameric repeats, the region on Chr 6 is the largest internal telomeric repeat in the genome and is associated with a break in assembly contiguity.

### Short-read coverage confirms contig assembly accuracy

**Fig. 4:**
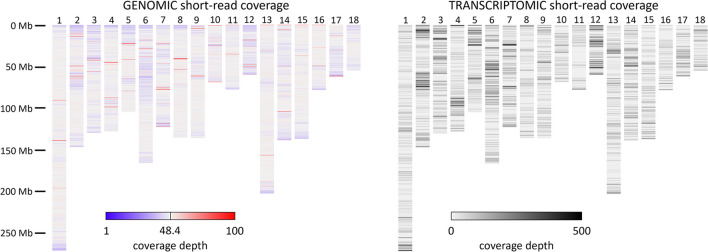
Genomic and transcriptomic short sequencing read coverage across TPI_Babraham_pig_v1. Genomic short sequencing reads from pig P18-11073 mapped to TPI_Babraham_pig_v1 and used to detect regions of anomalous coverage depth (*left*). The vast majority of regions displaying high coverage depth (shown in *red*) overlap the previously described centromeres, telomeres, and sequence gaps, while others reflect regions of high repetitiveness. Regions with less than average coverage depth (in *blue*) appear to largely overlap transcriptionally active regions of the genome (*right*). The transcriptome represented here is based on the combined coverage depth from all 13 tissues examined in the present study

Although sequence continuity may fail in the vicinity of large repeats, it is also possible that such regions could be erroneously collapsed or expanded while still assembling as a continuous sequence. We therefore mapped short reads from pig P18-11073 to the whole genome assembly to identify coverage anomalies that would indicate such errors. Mean genomic short-read coverage depth across the 18 autosomes was 48.4, which is very close to the expected value of 51. All regions with coverage greater than this were found to span relatively small distances (Fig. 4), the vast majority of which overlap the sequence gaps, centromeres, and telomeres, including the largest internal telomeric repeat on Chr 6. The highest coverage region not overlapping these features was found on Chr 4 circa base position 89.53 million and amid the Fc gamma receptor (FCGR) genes. There, the highest coverage positions were associated with long-interspersed nuclear elements (LINEs) and similar repeat elements found between the FCGR genes. Our recent annotation of this region in the Babraham assembly found that it is identical in gene content and organization to cattle, and Iso-Seq data from both species are consistent with their manually annotated intron–exon structures (Noble et al. 2023). Together, these observations strongly indicate that this region is correctly assembled. Low-coverage regions were found to be more broadly distributed, but with coverage depth never falling below 27.9 on average across 200 kb windows. Comparison with whole transcriptome short-read coverage from the same individual and time point indicates a large amount of overlap between transcriptomically active regions of the genome and lower-than-average genomic short-read coverage. Thus, we found no evidence of improperly assembled contigs based on short-read mapping.

### High homozygosity within the Babraham genome eases assembly

**Fig. 5:**
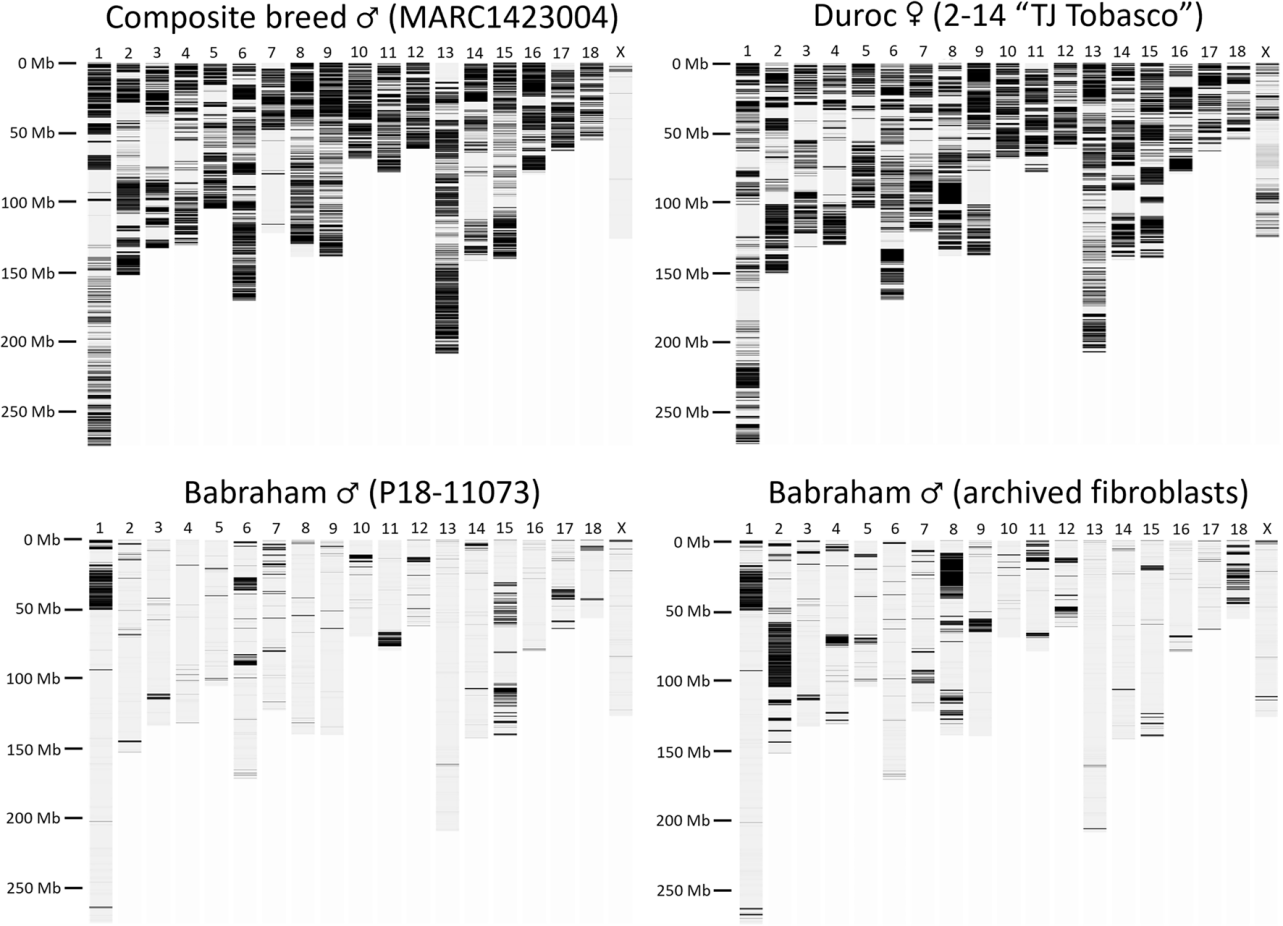
Heterozygosity of Babraham pigs and individuals used for the long-read pig genome assemblies. MARC1423004 was used to generate the USMARCv1.0 assembly; Duroc 2–14 “TJ Tabasco” was used to generate the Sscrofa11.1 assembly, and P18-11073 was used to generate the TPI_Babraham_pig_v1 assembly. The heterozygosity of a second Babraham individual is also shown (*lower right*) using whole genome sequencing reads generated from archival primary fibroblast cells. Reads from all individuals were mapped to Sscrofa11.1, and the number of heterozygous positions was summed and visualized using 200 kb sliding windows

Despite the ease of assembly and relatively modest amount of sequencing data used to generate the TPI_Babraham_pig_v1 assembly, it is 5.5 × more contiguous than USMARCv1.0 and 0.7 × as contiguous as the pig reference assembly, Sscrofa11.1 (Table 1). A high amount of homozygosity within the Babraham pig genome due to extensive inbreeding may in part explain the relatively high contiguity that was achieved. We therefore sought to assess the amount of heterozygosity across the Babraham genome, particularly as any remaining heterozygosity in the Babraham herd is expected to allow for phenotypic differences between individuals, possibly including immune-related traits. A total of 671,716 positions (0.030%) were heterozygous across the autosomes within the Babraham (P18-11073) Illumina sequencing reads (coverage depth, ~ 51 ×) (Table 2; Fig. 5). To compare between different Babraham individuals, genomic sequencing reads from the archived fibroblast cells of another male Babraham (coverage depth, ~ 28 ×) revealed 1,094,207 autosomal positions that were heterozygous (0.048%). These values contrast with the Duroc individual used to generate Sscrofa11.1 (Warr et al. 2020) in which 4,181,036 autosomal sites (0.185%) were heterozygous in that individual (coverage depth, ~ 46 ×). Likewise, sequencing reads from MARC1423004 (coverage depth, ~ 220 ×), the individual used to generate the USMARCv1.0 assembly, revealed 4,121,063 heterozygous autosomal positions. Thus, as expected given its history, the individual used to generate the Babraham pig genome is considerably more homozygous than either of the individuals used to generate the reference or the USMARCv1.0 assemblies.

**Table 2:** Heterozygosity of animals used in pig genome assembles

Chr	Number of heterozygous autosomal sites			
	Babraham P18-11073	Babraham fibroblasts	Duroc 2-14 TJ Tabasco	MARC 1423004
1	149,924	142,024	334,023	360,418
2	27,999	198,839	291,391	271,310
3	21,624	38,203	193,026	208,336
4	7347	59,090	177,950	207,559
5	12,018	31,223	233,828	198,982
6	78,645	26,059	295,567	340,965
7	47,860	43,725	221,471	125,536
8	12,361	217,931	295,207	302,047
9	14,057	44,393	307,968	295,318
10	35,812	14,295	193,232	218,003
11	52,966	64,659	188,629	169,332
12	24,031	48,358	140,636	171,210
13	11,038	16,926	348,306	350,704
14	32,986	19,045	264,190	192,907
15	91,850	32,739	278,352	247,408
16	5395	15,828	178,580	215,699
17	33,721	8818	150,041	146,865
18	12,082	72,052	88,639	98,464
Total	671,716	1,094,207	4,181,036	4,121,063

As a measure of autozygosity (i.e., identity by descent), runs of homozygosity longer than 1 Mb (ROH_1mb_) were identified by mapping the Babraham and Duroc Illumina reads to Sscrofa11.1. To determine an allowable density of heterozygosity to include in the ROH, a background error rate was calculated using the Babraham Chr X. Except for mapping and sequencing errors, Chr X from the male Babrahams should have few or no heterozygous sites outside the pseudoautosomal region (PAR), which comprises the first approximately 6.9 Mb (Skinner et al. 2013). Outside this PAR, the mean error rate was calculated using 200 kb windows as one heterozygous position in 20 kb from the Babraham Illumina data. For both the P18-11073 and the archived fibroblast sample, this error rate varied slightly across windows, such that an upper 95th percentile error rate was calculated as being approximately one heterozygous site in 5 kb. Using the lower threshold of one heterozygous site in 20 kb, expressed as a proportion, the ROH_1mb_ was calculated to be 0.47 (P18-11073) and 0.60 (archived fibroblasts) of the Babraham Chr X outside of the PAR. However, the upper threshold of one heterozygous site in 5 kb resulted in a more expected ROH_1mb_ of 0.94 for both individuals. Therefore, this higher error rate threshold was used to calculate the ROH_1mb_ segments across the autosomes. A total of 337 (P18-11073) and 325 (archive) ROH_1mb_ segments were identified across the Babraham autosomes, amounting to approximately 1971 Mb (87% of autosomal sequence) and 1836 Mb (81%), respectively (Table 3). In contrast, 189 ROH_1mb_ segments were identified in the Duroc autosomes totaling approximately 643 Mb, or 28% of the autosomal sequence, and for MARC1423004, the autosomes contained 155 ROH_1mb_ segments comprising approximately 554 Mb (22%). Thus, the Babraham pig displays a considerable amount of autozygosity due to intense inbreeding.

**Table 3:** Runs of homozygosity > 1 Mb in Babraham, Duroc, and MARC individuals

Chr	Number of ROH segments				Size of ROH (Mb)			
	Babraham P18-11073	Babraham fibroblasts	Duroc 2-14 TJ Tabasco	MARC	Babraham P18-11073	Babraham fibroblasts	Duroc 2-14 TJ Tabasco	MARC 1423004
1	30	34	25	20	226	223	101	94
2	19	20	14	16	136	80	44	48
3	18	16	12	13	122	116	53	56
4	13	17	10	12	124	107	59	40
5	16	19	7	7	96	79	33	21
6	27	24	11	13	141	158	35	31
7	25	19	11	7	100	101	35	78
8	23	23	12	7	127	75	29	19
9	19	12	9	8	132	122	44	11
10	14	15	4	2	62	63	7	3
11	10	7	6	3	65	59	17	4
12	15	11	4	1	53	46	9	2
13	28	32	17	15	199	196	42	39
14	24	26	12	7	130	134	40	55
15	23	19	11	11	90	125	33	24
16	11	11	9	5	72	70	23	14
17	15	13	7	4	47	57	18	7
18	7	7	9	4	49	26	21	7
X	20	24	14	7	112	116	38	120
1 to 18	337	325	190	155	1971	1837	643	554
Total	357	349	204	162	2083	1952	680	674

### Immune-related gene complexes are largely contiguous

Due to their repetitive nature, immune-related gene complexes are often poorly assembled in whole genome sequencing efforts. The nature of somatically rearranging B cell and T cell receptor genes also potentially complicates genome assemblies across these regions when using genomic DNA derived from blood. To mitigate this, we selected the largely immune-privileged cerebral cortex as a source of genomic material for the present study. Given the utility of the inbred Babraham pig for immunological studies, we sought to examine several immune-related genomic regions that are functionally important in lymphocyte immunobiology and commonly misassembled in whole genome sequencing efforts. As these regions tend to be largely complete in both the reference and the Babraham assemblies, we compared them to provide deeper understanding of the potential haplotypic diversity within these regions.

#### The T cell receptor (TCR) loci

**Fig. 6:**
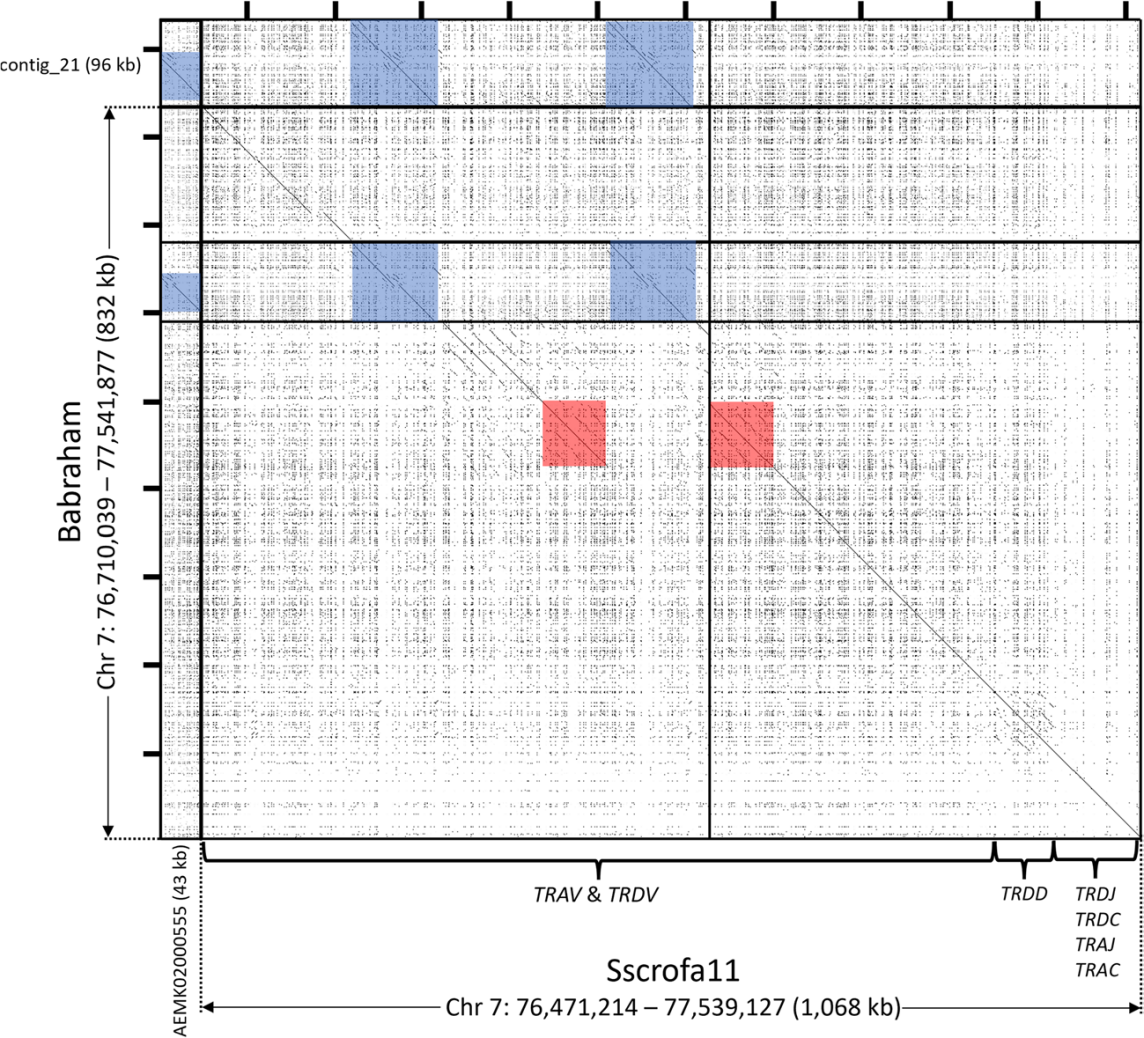
Contiguity and repetitiveness of the TRA/D locus. Recurrence plot comparison between the TPI_Babraham_pig_v1 (*vertical axis*) and Sscrofa11.1 (*horizontal axis*) assemblies. Gaps in the Babraham and Sscrofa assemblies are indicated by thick horizontal and vertical lines, respectively. Unplaced contigs in both assemblies are depicted here upstream from the V region. A ~ 73 kb region that is duplicated in the Sscrofa11.1 assembly, but not the Babraham, is shaded *red*, and a ~ 95 kb region that is duplicated in the Babraham assembly and triplicated in Sscrofa11.1 is shaded *blue*. Tick marks on top and at left are each separated by 100 kb

The pig TCR alpha and TCR delta chains are encoded within the same gene cluster, TRA/D (Babraham Chr 7: 76,710,039 – 77,541,877). This is the largest and most gene-dense region presently described, spanning approximately 1 Mb and containing approximately 118 *TRAV* and *TRDV* gene segments, and is rarely continuously assembled. Short-read mapping to the Babraham assembly also revealed abnormally high coverage in this region (Fig. 4), indicating potential issues in the assembly such as sequence gaps or misassembly. In the Babraham assembly, there are two sequence gaps and one 96 kb unplaced contig (contig_21). In Sscrofa11.1 (position: 7: 76,471,214 – 77,539,127) there is one sequence gap and a 43 kb unplaced contig (GenBank accession: AEMK02000555). Specific details regarding individual genes and polymorphisms are complicated by disruptions in the assemblies and the high similarity between many of the V gene segments. A ~ 73 kb duplication within the V region is present in Sscrofa11.1, but not the Babraham, and another ~ 95 kb duplication is found in both (Fig. 6). Peculiarly, these duplicated regions are not in the same locations in both assemblies. However, the actual organization is difficult to determine as the sequence gaps in both assemblies are all adjacent to these duplications (Fig. 6), thus implicating these duplications and their repetitiveness to the lack of continuity across the V region.

**Fig. 7:**
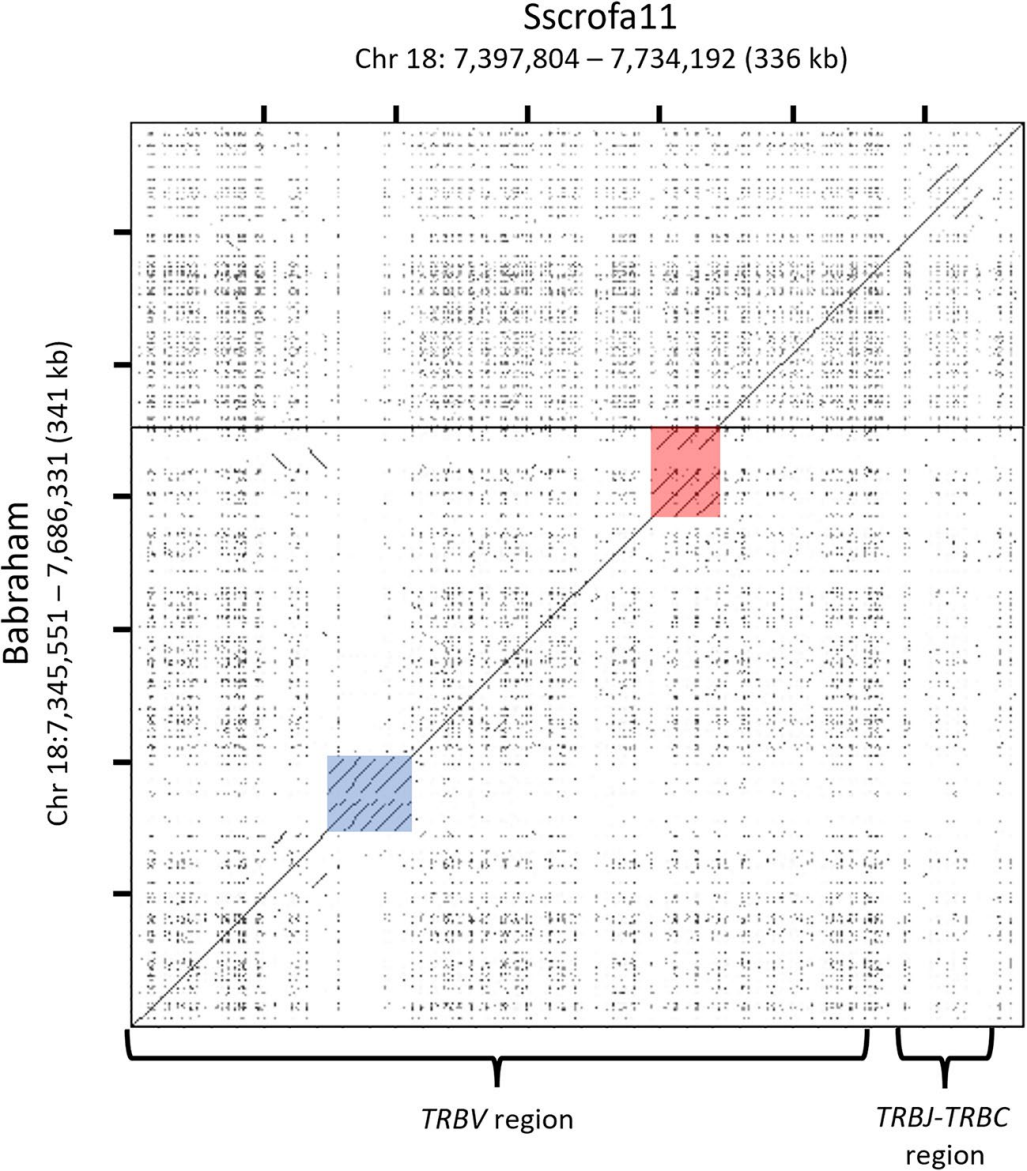
Contiguity and repetitiveness of the TRB locus. Recurrence plot comparison between the TPI_Babraham_pig_v1 (*vertical axis*) and Sscrofa11.1 (*horizontal axis*) assemblies. A single sequence gap in the TPI_Babraham_pig_v1 assembly — the only such gap on Chr 18 — is indicated as a thick horizontal line. This sequence gap is adjacent to a ~ 26 kb (Sscrofa11) to ~ 34 kb (Babraham) region containing three tandemly duplicated *TRBV* paralogs present in both assemblies (region shaded in *red*). In the Babraham, this region is larger due to an additional L1 insertion. Another ~ 32 kb region (shaded in *blue*) containing 10 closely related *TRBV* paralogs in Sscrofa11.1 appears to vary in gene content between haplotypes, as the same region only contains eight *TRBV* genes in the Babraham assembly

The TCR beta chain (TRB) region (Babraham Chr 18: 7,345,551 – 7,686,331) has been previously described for the Sscrofa11.1 assembly (Chr 18: 7,397,804 – 7,734,192) (Massari et al. 2018). Within that assembly, the *TRB* is intact on a single contig that spans the entire chromosome (~ 56 Mb), whereas a single sequence gap disrupts the *TRB* in the Babraham assembly — the only such sequence gap in the Chr 18 assembly. The Sscrofa11.1 *TRB* region contains 38 described *TRBV* genes (Massari et al. 2018) compared to 36 *TRBV* genes in the Babraham assembly. Recurrence plot analysis comparing the two assemblies revealed two distinct *TRBV* regions containing highly repetitive sequence (Fig. 7). Of these, the *TRBC*-distal region is variable in gene content containing ten *TRBV* genes in Sscrofa11.1 (*TRBV4-1* to *TRBV2-5*), but only eight in the Babraham. The *TRBC*-proximal region contains three highly similar *TRBV* genes (*TRBV20-1* to *TRBV20-3*) in both assemblies, plus an L1 insertion in the Babraham. This C-proximal cluster also abuts the Babraham sequence gap, and thus, the sequence similarity within this gene cluster presumably contributed to the disruption of the Chr 18 assembly.

The pig TCR gamma chain (TRG) region (Babraham Chr 9: 108,295,979 – 108,409,334) has recently been described in detail for the Babraham, Sscrofa11.1, and USMARCv1.0 assemblies (Le Page et al. 2021; Linguiti et al. 2022). In the Babraham, this region is intact and in the middle of a 41.7 Mb contig. The region contains four polymorphic V-J-C gene cassettes in both the Babraham and Sscrofa11.1 (Chr 9: 108,678,980 – 108,791,795) assemblies, although only three cassettes were identified in the USMARCv1.0 assembly (Chr 9: 30,653,846 – 30,739,227) (Le Page et al. 2021). Although the first of these cassettes was found to be the most abundantly expressed in general, *TRGV6* (of the second cassette) was previously found to be the single-most transcribed V gene segment, and while *TRGV6* is functional in the Babraham, it is putatively non-functional in the other porcine assemblies, due to being out-of-frame (Le Page et al. 2021).

#### The B cell receptor (BCR) loci

The immunoglobulin heavy chain (IGH) region (Babraham Chr 7: 125,292,945 – 125,642,007) is assembled to the telomeric end of Chr 7 on a 46 Mb contig, confirming previous cytogenetic evidence for its localization (Yerle et al. 1997). This region is unplaced in previous pig reference assemblies. Within Sscrofa11.1, the *IGH* region is split across at least six unplaced contigs (GenBank: AEMK02000149, AEMK02000151, AEMK02000188, AEMK02000452, AEMK02000566, and AEMK02000599); in particular, the Sscrofa11.1 IGH constant region and four *IGHV* genes are assembled to the end of a 3.8 Mb contig (GenBank: AEMK02000452). In pigs, this region is variable in *IGHG* content (and thus IgG isotypes). *IGHG1*, *IGHG3*, and *IGHG4* seem to be found in all haplotypes, whereas six additional *IGHG* genes have been found to be variably present depending on the haplotype (Zhang et al. 2020). The Babraham assembly itself contains *IGHG1*, *IGHG3*, and *IGHG4*, as well as *IGHG2a*, which is a close paralog of *IGHG4*. In contrast, the unplaced contiguous Sscrofa11.1 sequence contains the same four *IGHG* as the Babraham, in addition to *IGHG5a* and *IGHG2c*. A total of 25 *IGHV* gene segments, including 13 that are putatively functional, are present in the Babraham assembly. The *IGHV* gene most distal to the constant region sits a mere 4 kb from the telomeric end of the assembly, and since the flanking telomere is not present, the assembled *IGHV* region is possibly incomplete. A BLAST survey identified three additional small unplaced contigs (contig_547, 1.5 kb; contig_1142, 7.6 kb; and contig_1640, 29.1 kb) containing one, one, and four *IGHV* pseudogenes, respectively. These may represent either additional constant region-distal gene segments or alternative alleles that could not be assembled.

The immunoglobulin lambda light chain (IGL) region (Babraham Chr 14: 48,527,945 – 48,766,643) is continuous within a 15.2 Mb contig and falls within a 16 Mb ROH in both Babraham Illumina datasets. This region was previously characterized using overlapping BACs derived from the same Duroc individual used to generate the reference assembly, Sscrofa11.1 (Schwartz et al. 2012b). The IGL region is known to be polymorphic and possibly variable in gene content, as evidenced by *IGLV3-6* which can be present as either a null allele or as a highly transcribed functional allele (Guo et al. 2016; Schwartz and Murtaugh 2014). This diversity is apparent in the Babraham as well since both *IGLV3-6* and the adjacent *IGLV3-2* are deleted. The *IGLC* region likewise appears to be variable in gene content. The previous BAC characterization revealed three *IGLJ*-*IGLC* cassettes and *IGLJ4* with no corresponding downstream *IGLC* (Schwartz et al. 2012b). The IGL region within the Sscrofa11.1 assembly (Chr 14: 48,741,433 – 49,012,235), however, contains four intact cassettes, plus *IGLJ4*, and peculiarly the Babraham assembly contains six *IGLJ*-*IGLC* cassettes, as well as *IGLJ4*. In all assemblies, the most 5′ *IGLJ* contains the same non-canonical “FSGS” motif as described for *IGLJ1*, and the remaining cassettes all possess the same 1.3 kb spacing and canonical “FGGG” motif as described for the *IGLJ2* and *IGLJ3* gene segments, indicating that the more distal 3′ *IGLJ*-*IGLC* cassettes with canonical *IGLJ* are particularly prone to expansion and/or contraction.

The immunoglobulin kappa light chain (IGK) region (Babraham Chr 3: 57,436,231 – 57,625,777) is fragmented by two sequence gaps within the repetitive *IGKV* region. This includes a small (11.9 kb) intervening contig flanked by two much larger contigs containing the 5′ and 3′ ends of the region. This lack of contiguity is also reflected in the short-read mapping data as a region of abnormally high coverage (Fig. 4). In contrast, the same region in Sscrofa11.1 (Chr 3: 57,118,524 – 57,321,145) is continuous. As with the IGL, this region was previously characterized using BAC sequences derived from the same Duroc individual used to generate Sscrofa11.1 (Schwartz et al. 2012a). However, that characterization was incomplete, as it only identified the 14-most *IGKC*-proximal *IGKV* gene segments. We have therefore characterized the IGK gene content in both the Babraham and Sscrofa11.1 assemblies and identified 19 *IGKV* and 23 *IGKV* gene segments in the respective Chr 3 assemblies. In the Babraham, *IGKV2-13* and *IGKV1-14* appear to be missing in a sequence gap and a BLAST search of unplaced contigs did not identify them. However, unplaced contigs were identified in both assemblies which indicate that this region is considerably larger than the Chr 3 assemblies suggest. The unplaced contig AEMK02000525 in Sscrofa11.1 contains an additional 40 *IGKV* gene segments spanning 305 kb, and unplaced contig_369 from the Babraham assembly contains an additional 13 *IGKV* gene segments spanning 112 kb. Both unplaced contigs contain unique representatives from *IGKV* clan II, and allele sequences and organization of the *IGKV1* and *IGKV2* subgroups are distinct from those represented on Chr 3. This indicates that these contigs do not originate from the alternative haplotype and are likely best positioned within one of the Babraham sequence gaps. It is therefore apparent that due to the genomic complexity of the IGK locus, this region in Sscrofa11.1 was incorrectly continuously assembled, whereas in the Babraham assembly, it was disrupted by sequence gaps and additional contigs.

#### The leukocyte receptor complex (LRC)

The LRC (Babraham Chr 6: 58,236,196 – 58,935,786) is continuous in the Babraham assembly but disrupted in Sscrofa11.1 (Chr 6: 55,898,983 – 59,234,370) by the presence of a sequence gap and large inversion due to misassembly within a 197 kb sub-region that contains 17 repetitive leukocyte immunoglobulin-like receptor (*LILR*) genes and fragments from two distinct sub-families (Schwartz and Hammond 2018). In contrast, the Babraham assembly contains fewer *LILR* than Sscrofa11, with only 11 genes, including two gene fragments. Compared to our previous characterization of the LRC in Sscrofa11.1, the identified genes in the Babraham correspond to *LILR1B1* and *LILR2B8* to *LILR1A16*, with *LILR2B2* to *LILR1A7* being absent from the Babraham genome. Despite this, all six putatively functional genes in the Sscrofa11.1 assembly are also functional in the Babraham, and in addition to these, *LILR2B8*, which is putatively non-functional in Sscrofa11.1, is putatively functional in the Babraham. The remaining genes of the LRC, including the gene content variable novel immunoglobulin-like receptor genes, are similar to the described Sscrofa11.1 assembly (Schwartz and Hammond 2018). Genomic short-read variant calling failed to identify a single heterozygous site within the Babraham LRC.

#### The natural killer complex (NKC)

The Babraham NKC (Babraham Chr 5: 63,923,511 – 65,716,322) is continuous within a 23.4 Mb contig and within a > 5 Mb ROH in both Babraham Illumina datasets. Genomic short-read variant calling identified only one heterozygous site located in an intergenic region of the Babraham NKC (Chr 5: 65,428,924). This region is likewise contiguous within Sscrofa11.1 (Chr 5: 61,441,125 – 63,228,372) as previously described (Schwartz et al. 2017). The killer cell C-type lectin-like receptors (KLR) are represented by a minimal set of genes in the pig. This includes a single *KLRC* gene which is otherwise highly expanded in other species including bovids and equids. Humans, in contrast, have four *KLRC* genes (encoding NKG2A and -B, which are splice variants of *KLRC1*, and -C, -E, and -F) (Schwartz et al. 2017). Furthermore, we found no indication of gene content variation across this region between the two assemblies.

#### The major histocompatibility complex (MHC)

**Fig. 8:**
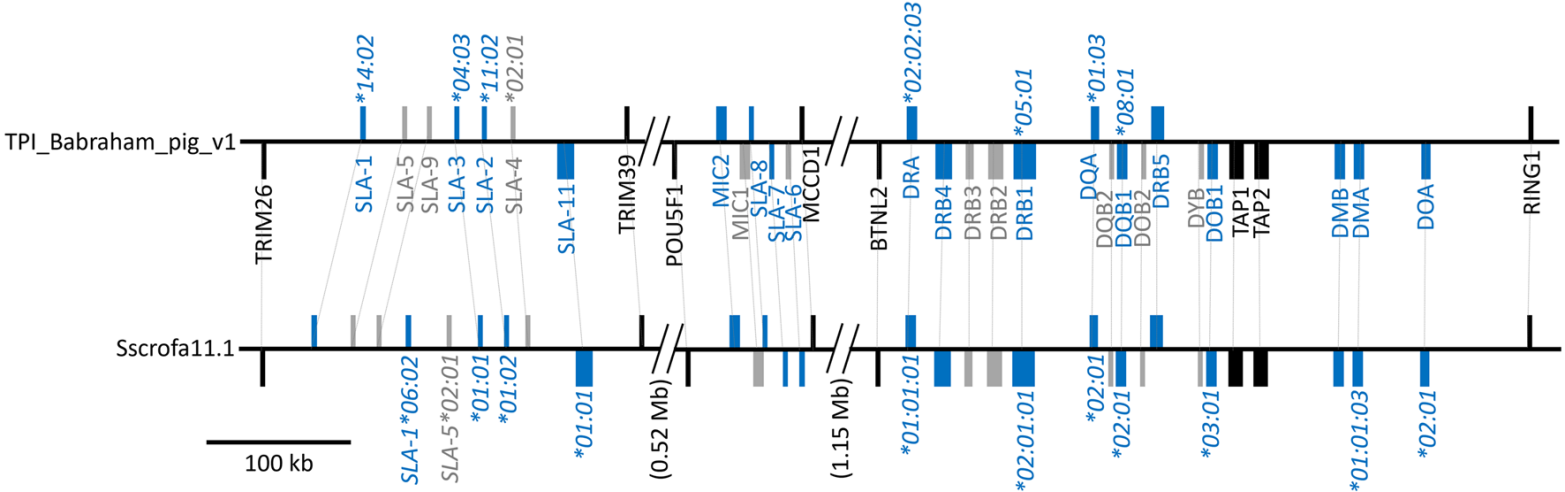
Organization of the MHC in Babraham pigs and the pig reference assembly, Sscrofa11.1. MHC class I and class II genes span three distinct regions on pig chromosome 7 which are separated by diagonal slashes and an open backbone. The distances between each of these regions are shown at the bottom. The centromere is positioned within the 1.15 Mb region separating the class I and class II regions. Colored vertical bars representing individual gene loci are shown to reflect their direction of transcription depending on whether they are oriented above (forward) or below (reverse) the horizontal backbone. Loci colored in blue represent putatively functional alleles, whereas those in grey represent putatively non-functional alleles and pseudogenes. Loci colored in black represent non-MHC genes and are included for positional reference. Curated alleles are shown with their official IPD-MHC designations next to their respective loci. Thin, dotted, grey lines indicate the same corresponding genes in the two assembled haplotypes, with the reference assembly having additional *SLA-1* and *SLA-5* loci. *SLA-6* is putatively functional in the reference assembly, but putatively non-functional in the Babraham assembly

The MHC class I region (Babraham Chr 7: 23,090,615 – 23,868,138) and the class II region (Babraham Chr 7: 25,057,296 – 25,415,322) are separated by the MHC class III region which includes the centromere and two associated sequence gaps. The organization of the Babraham MHC class I and class II regions are consistent with earlier characterizations of the pig MHC, including the Sscrofa11.1 assembly (Hammer et al. 2020; Renard et al. 2006), with some expected gene content variation among the classical class I genes (Fig. 8).

We previously determined that Babraham pigs are homozygous for the MHC haplotype Hp-55.6 (Schwartz et al. 2018), which is confirmed in the present assembly. In addition to the previously described alleles for *SLA-1*, *SLA-2*, and *SLA-3* within the classical MHC class I region, we further identified additional pseudogenes for *SLA-4*, *SLA-5*, and *SLA-9*, as well as functional *SLA-11* (Fig. 8). Moreover, *SLA-6* was found to possess a deletion encompassing all of exon 1, with no potential alternative leader exon identified. The designation of Babraham *SLA-6* as a null allele is consistent with our earlier finding that all cDNA sequences for *SLA-6* in five Babraham pigs were unspliced (Schwartz et al. 2018).

The MHC class II region is located approximately 180 kb from the centromere on the long arm of Chr 7. In addition to the described class II alleles for *SLA-DRB1* and *SLA-DQA* within the Hp-55.6 haplotype, we further determined the allele designations for *SLA-DRA* and *SLA-DQB1* (as shown in Fig. 8). Both *SLA-DRB4* and *SLA-DRB5*, while although generally considered pseudogenes and currently not represented within the Immuno Polymorphism Database (IPD)-MHC (Maccari et al. 2020), appear putatively functional in both the Babraham and Sscrofa11.1 assemblies, although future work is necessary to determine whether they are functionally transcribed and translated. Genomic short-read variant calling failed to identify any heterozygous sites within the Babraham MHC-I or MHC-II.

### A Babraham pig transcriptome atlas reveals tissue-specific gene expression

**Fig. 9:**
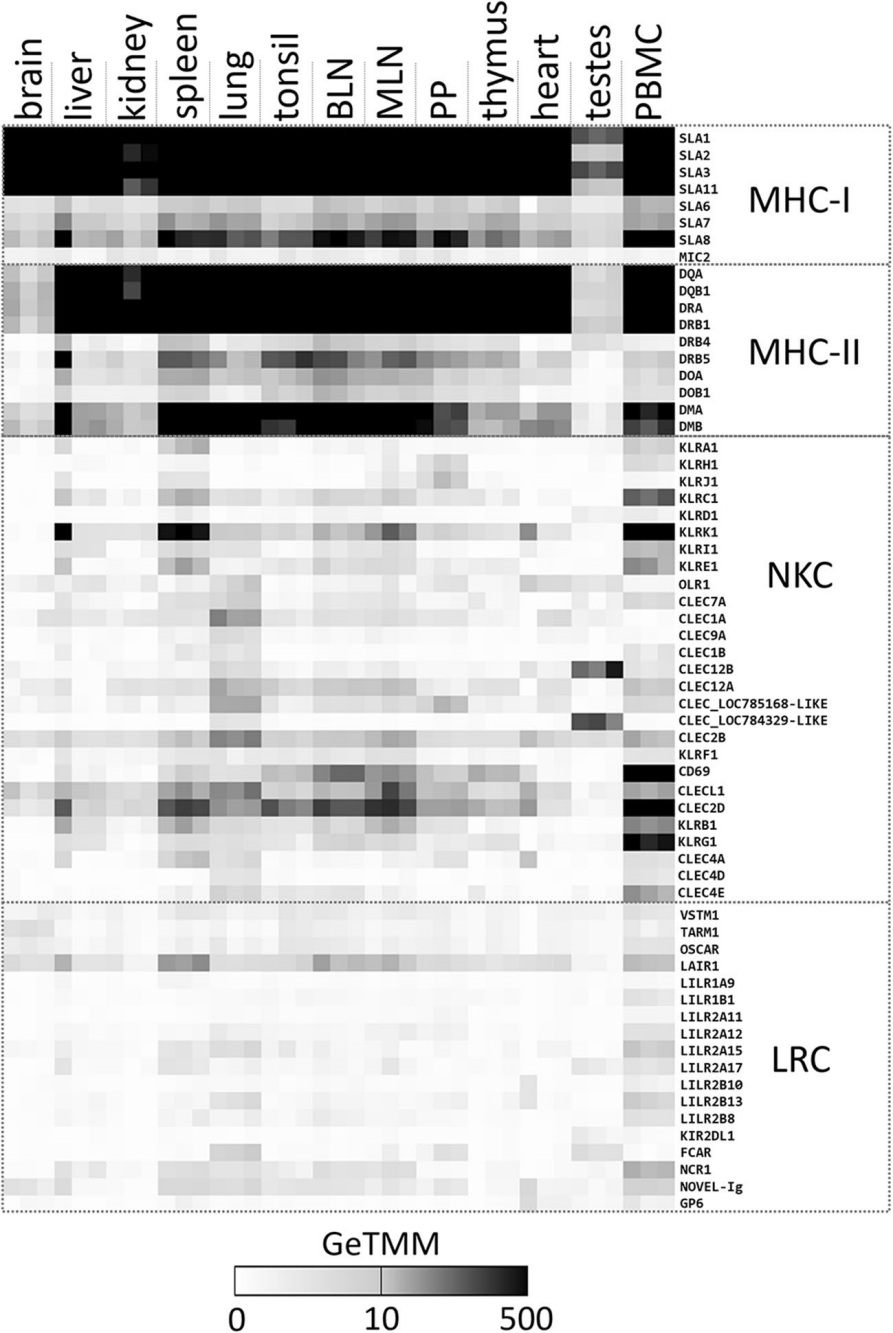
Transcriptomic expression of MHC class I and class II, NKC, and LRC genes in 13 tissues from pig P18-11073. All four gene complexes were found to be homozygous in the studied individual such that the expressed transcripts and haploid assembly contain the same alleles. Transcript counts were gene-length normalized to allow comparison between genes, and their trimmed mean of M-values were calculated (GeTMM) based on the number of reads mapping to annotated genes within the Sscrofa11.1 reference assembly. Babraham gene annotations shown here are based on the manual annotations reported herein and in previous reports (Schwartz et al. 2017; Schwartz and Hammond 2018). Each of the 13 tissues was processed and sequenced in triplicate as detailed in the “Materials and methods.” BLN, bronchial lymph node; MLN, mesenteric lymph node; PP, Peyer’s patch; PBMC, peripheral blood mononuclear cells

To increase the utility of the Babraham assembly, additional tissues from the same individual were harvested and stored in RNAlater, and triplicate subsamples were used for transcriptome sequencing and analyses. We focused these analyses on the MHC class I and class II genes, the C-type lectin-like genes of the NKC, and the immunoglobulin-like genes of the LRC. Our analyses above indicated that these regions are homozygous in the sequenced pig and their manual annotation confirmed that they are correctly assembled. Much of the results are as expected, for example, the classical class I MHC genes, *SLA-1*, *SLA-2*, and *SLA-3* are ubiquitously expressed and at higher levels than the non-classicals (Fig. 9). And although tissue-specific expression of MHC genes is not apparent, immune-related tissue expression is generally higher compared to tissues such as the brain or testes. However, we found higher than expected transcription levels for *SLA-6*, as only unspliced sequences were previously found for the putatively non-functional allele in the Babraham pig. Presumed heterodimeric partners such as the class II DQ, DR, DO, and DM as well as KLRI/E of the NKC show similar transcript expression and tissue distribution. However, KLRD (CD94) transcripts are almost non-existent, despite being expected to form heterodimers with KLRC. Although the function of KLRJ remains unknown, it is believed to interact with an unidentified heterodimeric partner (Schwartz et al. 2017). Transcripts for KLRJ were found predominantly in Peyer’s patch and spleen, similar to KLRH. Transcription of LRC genes was relatively weak, with the LILR predominantly transcribed in PBMCs and spleen (Fig. 9). Previously, and similar to current findings, no functional transcripts for KIR2DL1 were found in a pig PBMC transcriptome dataset (Schwartz and Hammond 2018). However, low transcription of this gene was detected in the testes, suggestive of an alternative non-immune function.

## Discussion

The presently described PacBio long-read Babraham pig assembly, error-corrected with Illumina short reads, is more contiguous (contig N50 = 34.9 Mb) than the initial Sscrofa11 PacBio assembly (contig N50 = 14.5 Mb) that was generated prior to gap filling which included the earlier sequencing data and Nanopore reads, and slightly less than the final Sscrofa11.1 assembly (Warr et al. 2020). Thus, the final TPI_Babraham_pig_v1 assembly represents an alternative high-quality pig genome assembly that is comparable to the reference assembly. This assembly adds to the available genomic resources for pigs, which include other biomedically important breeds such as the Göttingen minipig and the Ossabaw miniature pig, for which genomic sequences are also available (Heckel et al. 2015; Zhang et al. 2021). As a high-quality assembly, this new resource will facilitate comparative genomics analyses between pigs and provide insights into diversity within and between pig breeds. It will be immediately useful to research involving the Babraham pig as a biomedical model.

Divergent haplotypes can negatively affect an assembly’s contiguity due to their competition for assembly into a haploid representation of a diploid genome. Thus, homozygosity should aid whole genome assembly, and recent approaches have therefore sought to limit the effect that heterozygosity has on contiguity. This includes using individuals from genetically isolated and/or bottlenecked populations (Bickhart et al. 2017), or alternatively, employing methods such as trio-binning, which capitalize on the heterozygosity in offspring of genetically divergent parents to generate two distinct haploid assemblies (Bredemeyer et al. 2021; Koren et al. 2018; Low et al. 2020; Rice et al. 2020). It is therefore plausible that the extreme homozygosity of the sequenced Babraham individual contributed to the relatively high contiguity of the currently described assembly.

Advancements over the last decade in long-read sequencing technologies and improved scaffolding techniques have allowed for dramatic improvements in the contiguity of whole genome assemblies at a greatly reduced economic cost. The completion of the pig reference genome, Sscrofa9, in 2009 was the result of an extensive global effort which used 4 × to 6 × Sanger whole genome shotgun (WGS) reads mostly derived from the CHORI-242 BAC library (Archibald et al. 2010) and achieved a contig N50 of 54.2 kb with extensive manual finishing and gap filling. The reference was later updated to Sscrofa10.2 (contig N50 = 576 kb) with > 30 × Illumina GAII short-read WGS mostly based on CHORI-242 (Groenen et al. 2012) and recently updated to Sscrofa11.1 (contig N50 = 48.2 Mb) with 65 × WGS PacBio RSII reads, error-corrected with Illumina HiSeq 2500 WGS reads, and gap filled using both Oxford Nanopore and Sanger reads derived from CHORI-242 (Warr et al. 2020). Chromosome assignment of Sscrofa11.1 (and USMARCv1.0) scaffolds, which we also based the Babraham chromosomal assignments on, was itself initially based on the earlier Sscrofa10.2 assembly (Groenen et al. 2012), and ultimately on earlier physical mapping data (Humphray et al. 2007). Thus, any scaffolding errors present in the earlier reference assemblies, including contig ordering and orientation, would have carried through to the current pig genome assemblies, including for the Babraham. Efforts to generate gapless telomere-to-telomere genome assemblies are expected to resolve these issues in the future (Kalbfleisch et al. 2024; Nurk et al. 2022).

Chr Y is highly repetitive and predicted to be approximately 30 Mb in the pig (Skinner et al. 2016). As a result of the repetitiveness and difficulty in assembling it, Chr Y is often excluded from mammalian genome assemblies. In the Babraham assembly, Chr Y is incompletely assembled to a 5.5 Mb scaffold that is poorly contiguous compared to the rest of the Babraham assembly. Therefore, much of the Chr Y sequence is expected to be represented among the unplaced contigs. Despite this, there is less unplaced sequence overall in the Babraham assembly (~ 40 Mb) compared to either Sscrofa11.1 (~ 65 Mb) or USMARCv1.0 (~ 330 Mb). Since much of this unplaced sequence is also expected to derive from alternative haplotypes (Koren et al. 2018), the relatively low amount of unplaced sequence likely reflects the high homozygosity of the sequenced Babraham pig.

In 1999, restriction fragment fingerprinting suggested a similar level of homozygosity in the Babraham pig as inbred mice (Signer et al. 1999), and after multiple generations of continued inbreeding, extensive genome-wide homozygosity was further confirmed in 2016 from the SNP genotyping of five Babraham individuals (Nicholls et al. 2016). The extent of homozygosity and the remaining regions of heterozygosity identified in that study mirror our present findings using whole genome short-read data; in particular, relatively extensive tracts of heterozygosity remain in some, but not all, Babraham individuals on Chr 2 and Chr 8. Such genetic variation may contribute to the phenotypic variation between Babraham individuals; however, overall phenotypic variation is greatly reduced compared to other large pig breeds.

The TPI_Babraham_pig_v1 assembly is publicly available (ENA/GenBank: GCA_031225015.1) and is expected to be annotated with the Ensembl Genebuild pipeline and become available in a future Ensembl release. Nonetheless, automated annotation of tandemly duplicated genes, particularly rapidly evolving immune genes, often fails due to their repetitiveness and limited orthology with species like humans and mice. Hence, they require manual annotation to be accurate (Peel et al. 2022; Tørresen et al. 2019). Sequencing of individual MHC-I and MHC-II alleles indicates homozygosity across those regions in all animals sequenced so far (Schwartz et al. 2018). Our current analyses confirm this and indicate that the NKC, LRC, and likely the IGL are also homozygous in the sequenced animal. However, because these gene complexes tend to be highly repetitive, and thus notoriously difficult to accurately assemble and map using short-read data, some of the limited heterozygosity observed is likely the result of mismapping. Thus, due to inevitable short-read mapping errors, our results likely underestimate homozygosity to some extent.

The *LILR* genes are the most complex of the pig LRC and have undergone recent expansions, as evidenced by the presence of many highly similar and tandemly repeated genes. It is therefore highly plausible for *LILR* gene content variation to exist between different haplotypes. This gene content variation may explain why the Babraham has fewer apparent *LILR* genes compared to the Sscrofa11.1 assembly. The homozygosity across the LRC in the sequenced Babraham may have eased the assembly across this region into a single contig, while the heterozygous Sscrofa11.1 assembly was disrupted (Schwartz and Hammond 2018).

The pig *TRA/D* locus at approximately 1 Mb is similar in scale to the human (1 Mb) and dromedary camel (877 kb) (Massari et al. 2021), but substantially less than bovines (3.5 Mb) (Connelley et al. 2014). This locus is considerably larger than any of the other somatically rearranging T cell and B cell receptor genes, and due to the large (~ 73 kb and ~ 95 kb) repeat structures, it remains particularly challenging to completely assemble. In contrast, the IGH locus of the Babraham assembly possibly represents the first completely assembled porcine IGH region and is correctly assembled to the telomeric end of the long arm of Chr 7 (Yerle et al. 1997). Although it remains to be verified if all Babrahams share the same IGH haplotype, the sequenced individual possesses four *IGHG* genes, including the variably present *IGHG2a*. While not found in all pigs, the expressed IgG2a subclass has recently been shown to have strong Fc binding to NK cells, and strong effector functions, including complement-dependent cellular cytotoxicity, antibody-dependent cellular phagocytosis, and degranulation of NK cells (Paudyal et al. 2022). Although fragmented, the Babraham assembly indicates that the pig IGK region is substantially larger than either previous reports or the reference assembly otherwise suggest. Furthermore, *IGLV3-2* and *IGLV3-6* are deleted in the sequenced Babraham haplotype, and similar variation was previously shown to skew the expressed IGL repertoire in favor of different gene segments (Guo et al. 2016; Schwartz 2013; Schwartz and Murtaugh 2014).

Of the immune-related gene complexes that we examined, only the non-classical MHC genes and the NKC region appear to be fixed in gene content between pigs. This potentially extensive haplotypic variation across these regions could thus have profound effects on the expressed porcine immunome and variable immune phenotypes between individuals. Due to this genomic variability, the utility and availability of genomic resources matched to an experimental animal model, such as the Babraham pig, is worth considering during experimental design.

## Data Availability

The TPI_Babraham_pig_v1 genome assembly is available from ENA/GenBank under the accession GCA_031225015.1. Illumina and PacBio reads used to generate the assembly are available under the BioProject accession PRJNA1009406. Illumina reads generated from the archived Babraham primary fibroblast cells are available under BioProject accession PRJNA992241. Babraham pig transcriptome reads are available under BioProject accession PRJNA1098952. Specific allele sequences described in the text and manually annotated for the immune-related gene complexes in the Babraham assembly are available from the authors upon request. Babraham pigs are a UK national capability resource managed by The Pirbright Institute (Woking, UK). Individuals or groups seeking access to the Babraham pig herd are encouraged to contact https://www.animal.health@pirbright.ac.uk.
